# Asymmetric sampling in human auditory cortex reveals spectral processing hierarchy

**DOI:** 10.1371/journal.pbio.3000207

**Published:** 2020-03-02

**Authors:** Jérémy Giroud, Agnès Trébuchon, Daniele Schön, Patrick Marquis, Catherine Liegeois-Chauvel, David Poeppel, Benjamin Morillon

**Affiliations:** 1 Aix Marseille University, Inserm, INS, Inst Neurosci Syst, Marseille, France; 2 APHM, Hôpital de la Timone, Service de Neurophysiologie Clinique, Marseille, France; 3 Cleveland Clinic Neurological Institute, Epilepsy Center, Cleveland, Ohio, United States of America; 4 Department of Neuroscience, Max-Planck-Institute for Empirical Aesthetics, Frankfurt am Main, Germany; 5 Department of Psychology and Center for Neural Science, New York University, New York, New York, United States of America; Newcastle University Medical School, UNITED KINGDOM

## Abstract

Speech perception is mediated by both left and right auditory cortices but with differential sensitivity to specific acoustic information contained in the speech signal. A detailed description of this functional asymmetry is missing, and the underlying models are widely debated. We analyzed cortical responses from 96 epilepsy patients with electrode implantation in left or right primary, secondary, and/or association auditory cortex (AAC). We presented short acoustic transients to noninvasively estimate the dynamical properties of multiple functional regions along the auditory cortical hierarchy. We show remarkably similar bimodal spectral response profiles in left and right primary and secondary regions, with evoked activity composed of dynamics in the theta (around 4–8 Hz) and beta–gamma (around 15–40 Hz) ranges. Beyond these first cortical levels of auditory processing, a hemispheric asymmetry emerged, with delta and beta band (3/15 Hz) responsivity prevailing in the right hemisphere and theta and gamma band (6/40 Hz) activity prevailing in the left. This asymmetry is also present during syllables presentation, but the evoked responses in AAC are more heterogeneous, with the co-occurrence of alpha (around 10 Hz) and gamma (>25 Hz) activity bilaterally. These intracranial data provide a more fine-grained and nuanced characterization of cortical auditory processing in the 2 hemispheres, shedding light on the neural dynamics that potentially shape auditory and speech processing at different levels of the cortical hierarchy.

## Abstract 2

Capitalizing on intracranial data from 96 epileptic patients, this study precisely estimates the processing timescales along the cortical auditory hierarchy and reveals that an asymmetric sampling emerges in associative areas.

## Introduction

Contrary to the classic neuropsychological perspective, speech processing is now known to be distributed across the 2 hemispheres, with some models positing a leftward dominance for verbal comprehension and a rightward dominance for processing suprasegmental features, including aspects of prosody or voice processing [1]. The origin and function of lateralization continues to be vigorously debated, for example, with regard to its domain-general or domain-specific nature [2,3]. The former view predicts that lateralization of speech processing (and auditory processing, in general) originates in general-purpose mechanisms sensitive to the low-level acoustic features present in speech. The domain-specific view postulates that speech is processed in a dedicated system lateralized to the left hemisphere. On this view, processing critically depends on the specific linguistic properties of a stimulus. Crucial to this debate is thus proper understanding of the distinctive sensitivity of the left and right auditory cortical regions to acoustic features, which should be grounded in characteristic anatomic-functional signatures.

There exists suggestive neuroanatomical evidence for structural differences between the left and right auditory cortex. The primary auditory cortex (A1, BA41) is larger in the left hemisphere, with a higher density of gray and white matter [4]. Moreover, the left auditory cortex contains larger cortical columns with a higher number of large pyramidal cells in cortical layer III than its right counterpart [5]. Those differences in cytoarchitectonic organization should coexist with electrophysiological and functional differences between auditory regions. Building on such observations, the asymmetric sampling in time (AST) hypothesis made several interrelated predictions related to the characteristics of auditory information processing at the cortical level [6]. The main tenets of the original AST hypothesis regarding anatomofunctional specifications can be synthetized as follows:

1. The human auditory system employs (at least) a two-timescale processing mode, characterized by oscillatory cycles that can be viewed as individual computational units. These 2 timescales operate in the low-gamma (around 25–50 Hz) and theta (around 4–8 Hz) frequency ranges, corresponding, respectively, to temporal integration windows of approximately 30 ms and 200 ms. Such temporal multiplexing allows the system to process in parallel acoustic information using 2 complementary algorithmic strategies, optimized to encode complementary spectrotemporal characteristic of sounds. This prediction—that sounds are processed at preferred and specific timescales—has received support from both auditory and speech-specific paradigms [7–15].
2. This dual-timescale processing operates in both hemispheres, but the ratio of neural ensembles dedicated to the processing of each timescale differs between left and right hemispheres. Indeed, while the left auditory cortex would preferentially process auditory streams using a short temporal integration window (30 ms), the right auditory cortex would preferentially sample information using a long temporal integration window (200 ms). Previous findings reported that left and right cortical auditory regions exhibit differences in their intrinsic oscillatory activity [16–18]. A relative leftward dominance of low-gamma neural oscillations and/or rightward dominance of theta oscillations is also visible during sensory stimulation [17,19,20]. This asymmetry is, moreover, reflected in the sensitivity of the left and right auditory cortex to different spectrotemporal modulations of sounds, with a leftward dominance for fast temporal modulations and/or a rightward dominance for slow temporal modulations [12,21–28].
3. The electrophysiological signature of this asymmetry emerges outside of primary auditory regions. The AST hypothesis in its original conception posited that at the level of core auditory cortex, there is no obvious functional asymmetry, but that beyond this first stage of cortical processing, a functional asymmetry should be visible, namely in the left and right association auditory regions. This last point has also received some empirical support [12,24,25,27].

While each of these predictions has received experimental support, they are also vigorously debated. In particular, one concern relates to the specificity of the left temporal lobe for faster temporal modulations. Some authors have suggested that most published results can be interpreted in an alternative framework, wherein only the right temporal lobe shows a marked preference for certain properties of sounds (for example, longer durations or variations in pitch [3,29]). Moreover, in contrast with the AST hypothesis, some authors suggested that the hemispheric asymmetry may stem from core auditory areas (Heschl’s gyrus) and not association cortex [16–18,21,22,30–32]. The conflicting results may be due to differences in paradigms and stimuli, as well as the resolution of the imaging instruments employed. However, anatomical and lesion studies also indicate hemispheric asymmetries at the level of the primary cortex [5,33]. Discrepancies may thus also arise from the fact that this asymmetry probably takes different forms along the auditory cortical pathway, with a more subtle functional signature at early cortical stages and more striking qualitative differences at later processing stages (see, for example, [17]). Finally—and this is a crucial aspect of the theory—the duration of these temporal integration windows was never precisely characterized physiologically with high-resolution data.

To more sensitively test the predictions of the AST hypothesis and overcome some of the difficulties in acquiring decisive data to characterize the signature of auditory hemispheric lateralization at both high spatial and temporal (hence also spectral) resolutions, we combined 2 innovative experimental approaches to noninvasively map the dynamical properties of distinct cortical auditory areas. 1) The brain is often described as a dynamical system oscillating at multiple frequencies [34,35]. Previous work has envisioned the event-related potential (ERP) as the impulse, stereotyped response of the brain. In such a framework, the dynamical properties of neuronal responses to external perturbations is assumed to depend both on the fined-grained structural constraints of the selective brain region investigated and on the characteristics of the stimulus [36,37]. Probing the brain with a short acoustic transient or impulse—i.e., a signal without any temporal dynamics—and examining the spectrotemporal properties of its evoked response therefore unveils the intrinsic dynamics of the auditory areas investigated. This method corresponds to a noninvasive mapping of the dynamical properties of specific cortical microcircuits [36]. 2) Thanks to the granularity offered by human intracranial recordings, a systematic investigation of the stereotyped response evoked by a brief (30-ms) pure tone can be performed in distinct cortical auditory areas. In addition to revealing the intrinsic dynamics of each region, it allows characterizing the functional asymmetry along the auditory pathway with a high spatial resolution. In the present study, we capitalize on data acquired from 96 epileptic patients, implanted for clinical evaluation at various stages of the auditory cortical hierarchy. Our results show the natural spectral profile of neural activity in left and right primary, secondary, and association cortical auditory regions, thus enabling a detailed characterization of the potential interhemispheric functional differences and dynamics at play during auditory processing.

## Results

**Fig 1:**
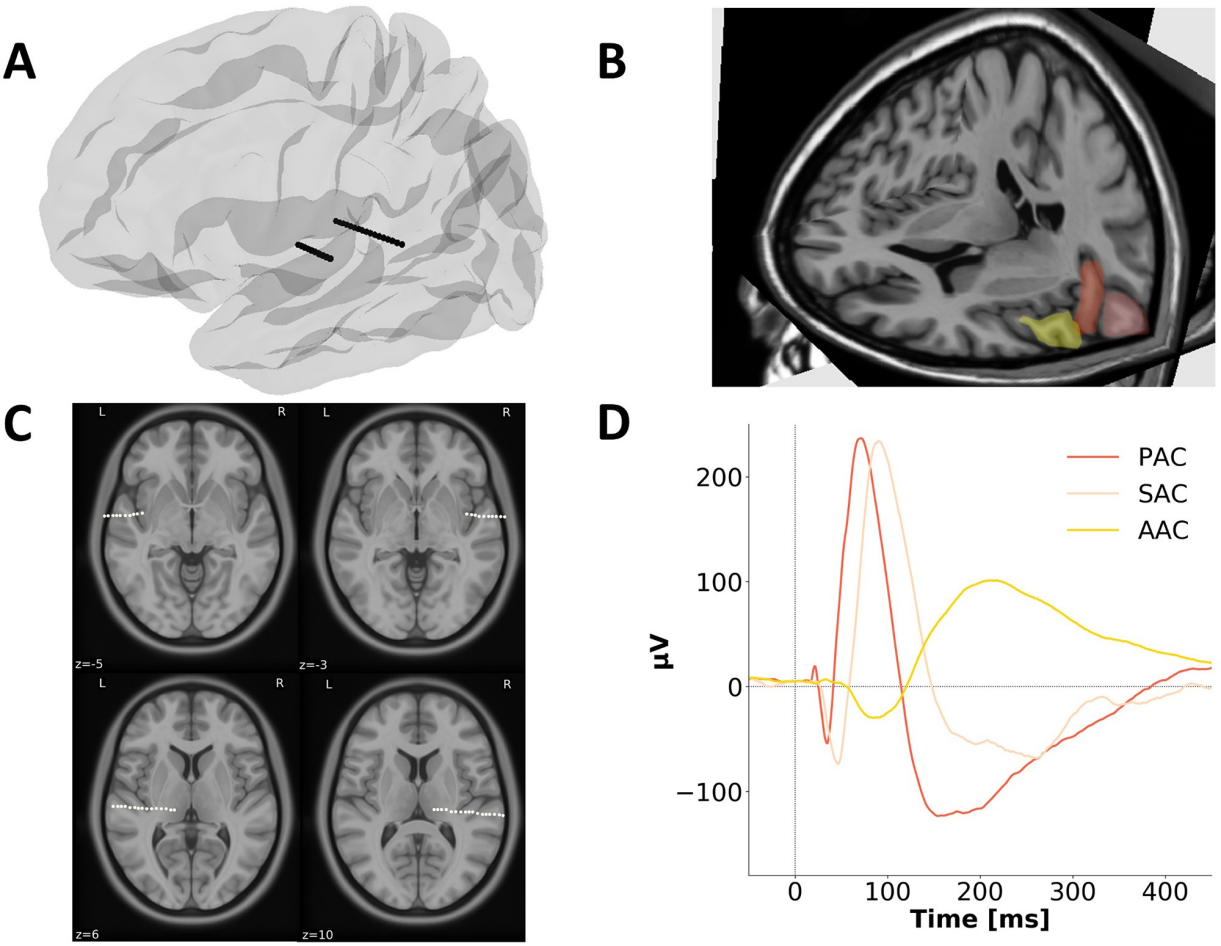
Example of electrodes and contact positions with characteristic AEPs in exemplar patients. (A) Schematic example of typical SEEG recording electrodes shown in a 3D view of the left hemisphere on a template brain in an MNI space. The posterior electrode is composed of 15 contacts and targets core auditory regions, while the anterior electrode, composed of 5–15 contacts, targets association auditory regions. (B) MRI scan showing the location of the 3 ROIs in the left hemisphere. Red: PAC, the posteromedial portion of Heschl’s gyrus (A1, medial belt and lateral belt areas; see [43]); pink: SAC, the lateral posterior STG (parabelt area); yellow: AAC, the lateral anterior STG (area A4). (C) Representative examples of SEEG recording electrodes shown in an axial view of a template brain in an MNI space, targeting left or right association (upper panels) or core (lower panels) auditory regions. (D) AEPs in response to pure tones from a representative patient for each ROI. The axes are color-coded according to the locations displayed in B. Electrode contacts used along the shaft were selected based on their anatomical location and functional responses (typical shape and latencies of evoked responses; see Materials and methods). AAC, association auditory cortex; AEP, auditory evoked potential; MNI, Montreal Neurological Institute; PAC, primary auditory cortex; ROI, region of interest; SAC, secondary auditory cortex; SEEG, stereotactic electroencephalography; STG, superior temporal gyrus.

Data from 96 epileptic patients implanted with depth macroelectrodes located in left and right primary, secondary, and association auditory cortex (PAC, SAC, and AAC, respectively) were analyzed (Fig 1) [38–41]. Auditory areas were defined with a functional localizer [30,38,39,42]. PAC, SAC, and AAC, respectively, correspond to the posteromedial portion of Heschl’s gyrus (A1, medial belt and lateral belt areas; BA41, anterior to Heschl’s sulcus), the lateral posterior superior temporal gyrus (STG; parabelt area; anterior portion of BA42, posterior to Heschl’s sulcus), and the lateral anterior STG (area A4; anterior BA22) [40,43]. 78% of the patients had a typical language lateralization in the left hemisphere (see Materials and methods). Left hemisphere dominance for language is usually observed in approximately 90% of healthy individuals and in 70% of epileptic patients [44]. Patients participated in a perceptual experiment during which they passively listened to pure tones and syllables (see Materials and methods).

### Spectral characteristics of the evoked response to transient pure tones

**Fig 2:**
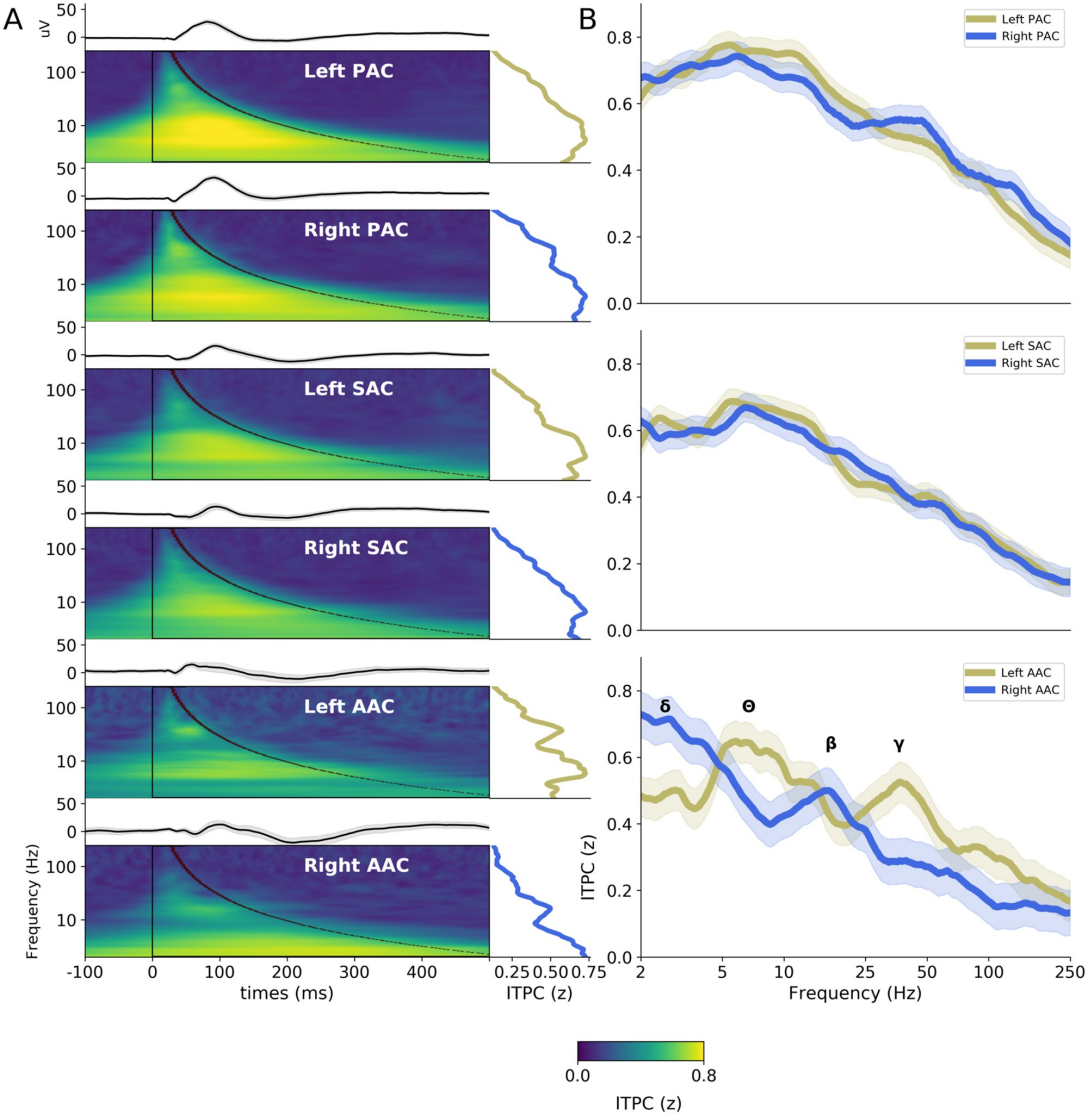
Evoked activity in response to pure tones (0.5 kHz and 1 kHz merged) in hierarchically organized auditory areas. (A) AEPs (top panels) and ITPC (lower panels) in response to pure tones, averaged across patients, in the 6 auditory ROIs (PAC, SAC, and AAC, in left and right hemispheres). Right insets indicate the spectrum of the ITPC averaged over one oscillatory cycle (black overlay). (B) Interhemispheric comparison of the ITPC spectra in PAC, SAC, and AAC. Shaded areas indicate SEM. Greek letters indicate the main peaks observed in AAC (δ: delta 1–4 Hz; θ: theta 4–8 Hz; β: beta 14–30 Hz; γ: low-gamma 25–45 Hz). Number of patients recorded at each location: left PAC = 39, right PAC = 27; left SAC = 40, right SAC = 29; left AAC = 12, right AAC = 12. AAC, association auditory cortex; AEP, auditory evoked potential; ITPC, intertrial phase coherence; PAC, primary auditory cortex; ROI, region of interest; SAC, secondary auditory cortex.

To investigate the fine-grained temporal constraints of the first cortical stages of the auditory processing hierarchy, we first analyzed the evoked, stereotyped (i.e., identical across trials) responses to transient acoustic impulses (30-ms duration pure tones, presented at 0.5 or 1 kHz). A time-frequency representation of the evoked responses, as computed through intertrial phase coherence (ITPC; Fig 2A), demonstrates the presence of a dynamical response composed of multiple spectral modes (i.e., time constants), which, moreover, could differ between regions of interest (ROIs). These responses were limited in time and homogenous, and their spectral profile was best captured by averaging ITPC values over time (see Materials and methods).

In PAC and SAC, a group-level analysis (computed after normalizing individual ITPC spectra to minimize the potential impact of a few outliers on group results) revealed the presence of a simple evoked response profile, characterized by a main spectral maximum within the theta range (around 4–8 Hz; corresponding to a time constant of approximately 150 ms; Fig 2B). Importantly, this response profile was similar across left and right hemispheres. Conversely, a more complex pattern of response was visible in AAC, with the presence of 2 distinct salient spectral maxima that moreover differed between left and right hemispheres. Prominent ITPC peaks in the theta (4–8 Hz) and low-gamma (25–50 Hz) frequency ranges were visible in left AAC; the right counterpart was characterized by peaks in the delta (1–4 Hz) and beta (13–30 Hz) frequency ranges (Fig 2B).

**Fig 3:**
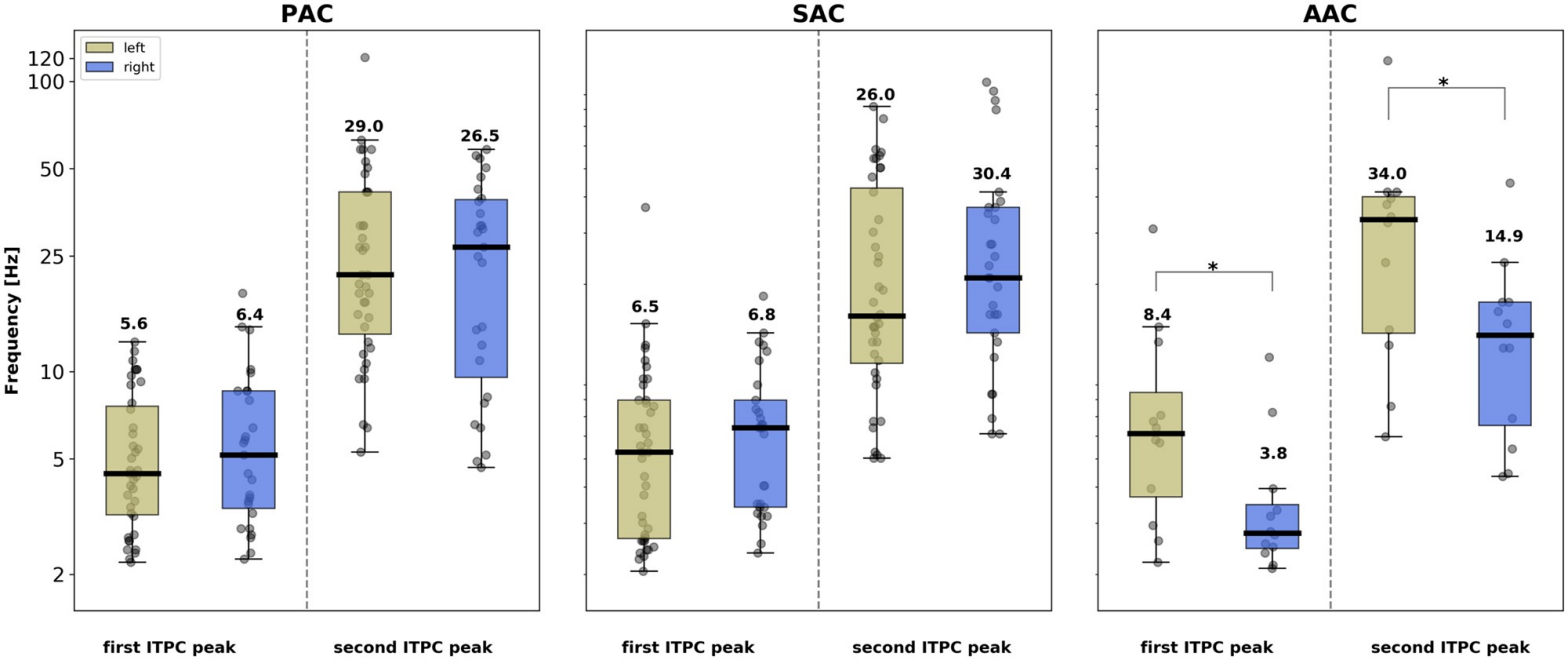
Interhemispheric comparison of the frequency of the 2 main ITPC peaks from the evoked response to pure tones (0.5 kHz and 1 kHz merged) in the different auditory areas. Individual peaks were identified as the 2 highest noncontiguous maxima of the ITPC spectrum (between 2–250 Hz). Frequency (in Hz) is denoted on the y-axis. Box plots: contour lines reflect the first and third quartile of the distribution, and the thick central line denotes the median. Numbers indicate the mean. Gray dots correspond to individual data. Stars indicate significant interhemispheric differences (unpaired Mann–Whitney U Tests, *p* < 0.05). AAC, association auditory cortex; ITPC, intertrial phase coherence; PAC, primary auditory cortex; SAC, secondary auditory cortex.

To better characterize the time constants of the neural processes occurring at each putative step of the auditory cortical hierarchy, we extracted for each patient and ROI the 2 highest local maxima of the ITPC spectrum (between 2–250 Hz; Fig 3 and S1 Fig). This analysis substantiates the finding that in PAC and SAC, the evoked response was dominated by an ITPC peak in the theta range (around 4–8 Hz) and highlights that a secondary peak emerged in the beta/gamma range (around 15–40 Hz; see interindividual spectral distributions in Fig 3). At these earlier cortical stages, the frequency of the 2 main ITPC peaks did not differ significantly across hemispheres, neither in PAC (Mann–Whitney U test: first peak, U = 485.0, *p* = 0.29; second peak, U = 496.5, *p* = 0.35) nor in SAC (first peak, U = 484.0, *p* = 0.12; second peak, U = 502.0, *p* = 0.17). In contrast, in AAC, a more complex and significantly asymmetric response profile emerged. We confirmed that the evoked response was characterized by higher-frequency peaks in left than right AAC, with respectively theta/gamma (around 8/35 Hz) peaks in left and delta/beta (around 4/15 Hz) peaks in right AAC. Interhemispheric comparison of the frequency of the 2 main ITPC peaks confirmed that this asymmetry was significant (first peak, U = 34.5, *p* < 0.05; second peak, U = 36.5, *p* < 0.05). To confirm the robustness of these findings, we reanalyzed the evoked response to pure tones separately for 0.5 and 1 kHz pure tones and observed the exact same ITPC spectral profile for each ROI, independently of the frequency of the pure tone (S2 Fig).

### Interindividual consistency of the spectral response profile in left and right AAC

**Fig 4:**
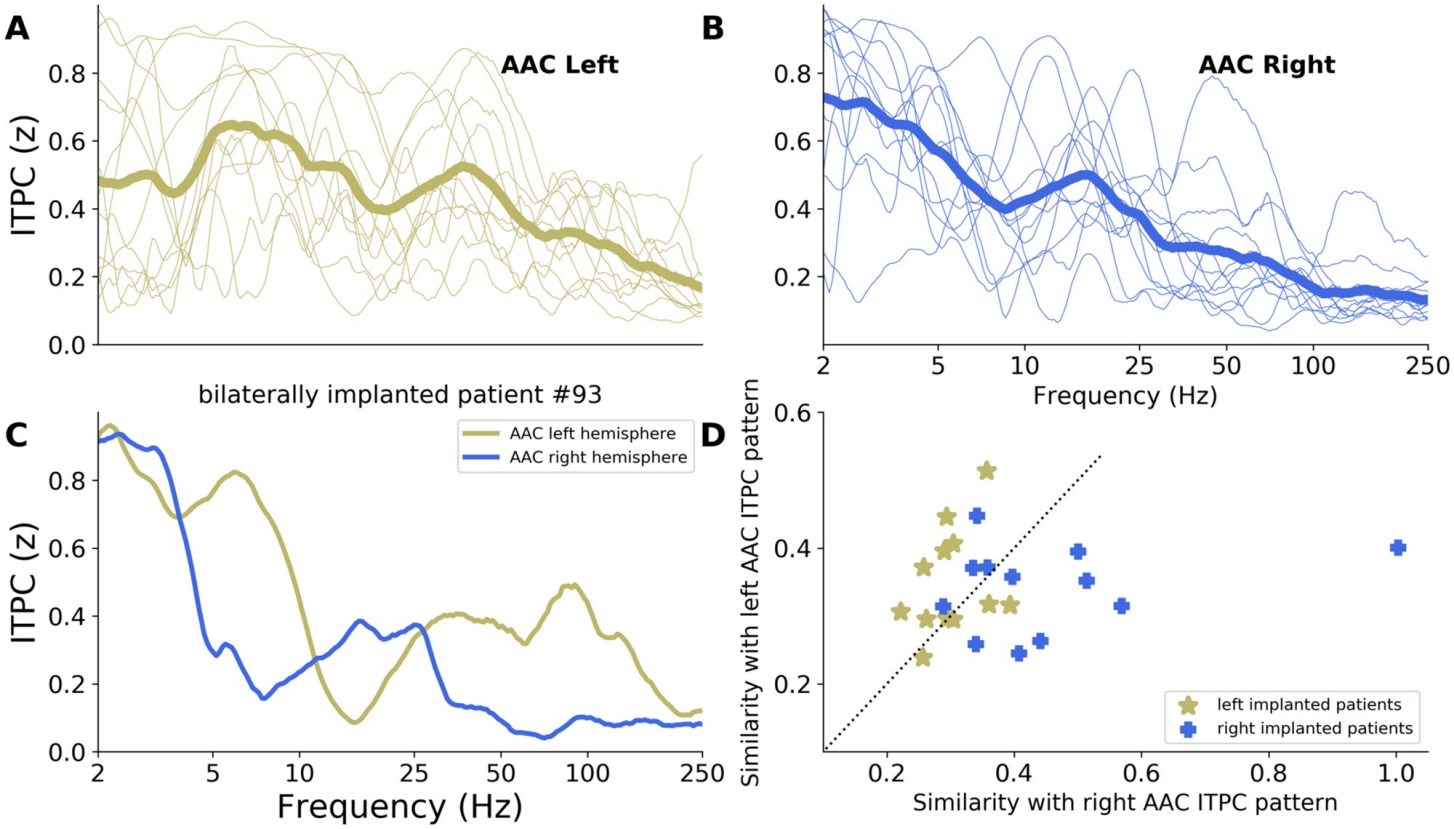
Interindividual consistency of the ITPC spectra in response to pure tones in left and right AAC. (A–B) Individual ITPC spectra in response to pure tones (0.5 kHz and 1 kHz merged). Thick lines: group-level spectral pattern. Thin lines: individual data (A: left AAC, *n* = 12; B: right AAC, *n* = 12). (C) ITPC spectra of a patient implanted bilaterally in AAC. (D) Similarity of each individual ITPC spectrum with the group-level ITPC patterns obtained in left (y-axis) and right (x-axis) AAC (see Materials and methods). The dashed diagonal indicates equal similarity to both group-level ITPC patterns. AAC, association auditory cortex; ITPC, intertrial phase coherence.

While our previous analyses were focusing on the frequency of the 2 main ITPC peaks, we next investigated hemispheric asymmetry while taking advantage of the entire ITPC spectrum (between 2–250 Hz). A leave-one-out cross-validation (LOOCV) procedure was used to assess the similarity of each individual ITPC spectrum with the left and right ITPC patterns obtained at the group level (Fig 4A and 4B). The rationale of this analysis is that group-level ITPC patterns are a good approximate of a prototypical response and can thus be used as “models” upon which individual data can be compared. Briefly, for each individual ITPC spectrum, we estimated its mean squared error (MSE, i.e., the error of fit) relative to both left and right AAC models (i.e., group-level ITPC patterns; see Materials and methods). On average, left AAC ITPC spectra were more similar to the left AAC model than the right one (unpaired Mann–Whitney U test: U = 36.0, *p* = 0.020). On the contrary, right AAC ITPC spectra were more similar to the right than the left AAC model (U = 40.0, *p* = 0.034). Moreover, the only patient implanted bilaterally in AAC showed an asymmetric response profile compatible with the group-level ITPC spectra (Fig 4C).

Then, we investigated whether this asymmetric response in AAC, visible at the group level, was robust at the individual level (Fig 4D). This methodology aims at determining whether the implantation hemisphere (left or right) of a patient can be predicted from the functional response of its AAC region to a brief auditory stimulation. This analysis revealed that for 16 out of the 24 (67%) patients implanted in AAC, their ITPC spectrum was more similar to the model (i.e., group-level ITPC pattern) of their hemisphere of implantation than to the one of the opposite hemisphere.

Amongst the 24 patients implanted in AAC, 4 had a nontypical language lateralization that was moreover complex, reflecting a bilateral organization of language functions. In a following analysis, we thus excluded them and recomputed the similarity analysis for the remaining 20 patients with a typical language lateralization in the left hemisphere. We observed that 14/20 (70%) patients had a spectral profile of response congruent with the ITPC pattern of their hemisphere of implantation (7/10 in left AAC, 7/10 in right AAC).

### Prototypical ITPC spectral components of the AAC response

Next, we analyzed the entire set of electrode contacts implanted in AAC. On the one hand, this approach allows evaluating the potential impact of our selection criteria (restricted to the electrode contact with the largest auditory evoked potential [AEP] per patient) on the previous results. On the other hand, having a large number of recordings is well-suited to perform data-driven analyses. All contacts from all electrodes implanted in left and right AAC were regrouped and analyzed as previously to extract their ITPC spectra in response to pure tones. A non-negative matrix factorization (NMF) was then conducted on this extended data set. This unsupervised clustering method allows extracting the prototypical ITPC spectral components constituting this data set, in which left and right AAC were combined (see Materials and methods).

**Fig 5:**
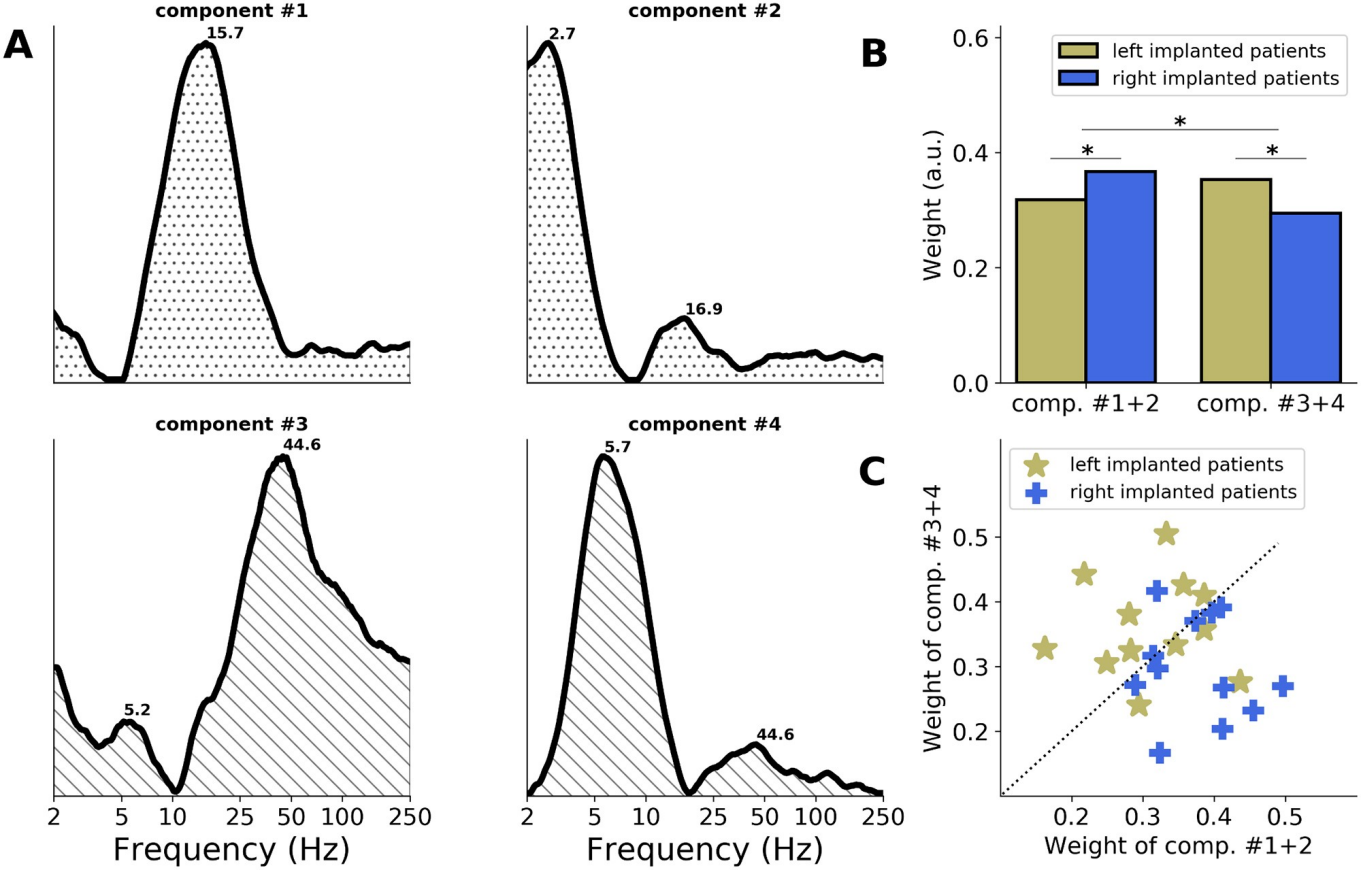
Main spectral components of the AAC response to pure tones. The NMF method applied to the ITPC spectra in response to pure tones (0.5 kHz and 1 kHz merged) of the entire set of electrode contacts implanted in AAC (left and right combined; *n* = 24). (A) Four main ITPC spectral components estimated with the NMF (see Materials and methods). Numbers indicate the main frequency peaks of each component. Components can be regrouped according to their spectral profile, into “delta/beta” (around 2.5/16 Hz; components #1 and 2) and “theta/gamma” (around 5/45 Hz; components #3 and 4) patterns. (B) Respective contribution (relative weight) of components #1 and 2 and #3 and 4 to left and right AAC data. Stars indicate significant differences (unpaired *t* tests, *p* < 0.05). (C) Respective contribution (relative weight) of components #1 and 2 (x-axis) and #3 and 4 (y-axis) to individual data. Patients are sorted according to the hemisphere of implantation (yellow: left; blue: right). The dashed diagonal indicates equal weights. AAC, association auditory cortex; ITPC, intertrial phase coherence; NMF, non-negative matrix factorization.

This analysis yielded 4 main ITPC spectral components, which altogether explained 63% of the variance of the data set. These components could be regrouped according to their spectral profile. Two components (#1 and #2) had main peaks in the delta (around 2.5 Hz) and/or beta (around 16 Hz) bands, and the other 2 components (#3 and #4) had main peaks in the theta (around 5 Hz) and gamma (around 45 Hz) bands (Fig 5A). This indicates that across the entire set of electrode contacts present in (left and right) AAC, the evoked responses to a brief acoustic stimulation are principally composed of delta/beta and theta/gamma bimodal spectral patterns.

We evaluated the relative weight of these components on left or right AAC responses (Fig 5B). Across electrode contacts, we observed a significant components by hemisphere interaction (unpaired *t* test: *t* = 4.3, *p* < 0.001), with components #3 + 4 (theta/gamma pattern) being significantly more present in left than right AAC (*t* = 3.6, *p* < 0.001), and components #1 + 2 (delta/beta pattern) being significantly more present in right than left AAC (*t* = 3.2, *p* = 0.002). To estimate whether this interaction was robust at the individual level, we extracted the contribution (relative weight) of each component to the individual data by averaging intrapatient electrode contacts (Fig 5C). We observed that the responses of 8/12 (67%) patients implanted in the left hemisphere were predominantly composed of the theta/gamma components (#3 and 4), while the responses of 10/12 (83%) patients implanted in the right hemisphere were predominantly composed of the delta/beta components (#1 and 2).

We finally replicated this analysis on the 20 patients implanted in AAC that had a typical language lateralization in the left hemisphere. We observed that 16/20 (80%) patients had a response profile predominantly composed of the expected spectral components (7/10 dominated by theta/gamma components [#3 and 4] in left AAC, 9/10 dominated by delta/beta components [#1 and 2] in right AAC). This new analysis (clustering of entire set of electrode contacts) thus reveals that the specificity of the spectral responses in left and right AAC is actually quite robust at the individual level. Overall, this confirms the existence of a functional asymmetry in AAC, with a predominant theta/gamma (around 5/45 Hz) activity in left AAC and a predominant delta/beta (around 2.5/16 Hz) activity in right AAC.

### Interaction between stimulus and neural dynamics

**Fig 6:**
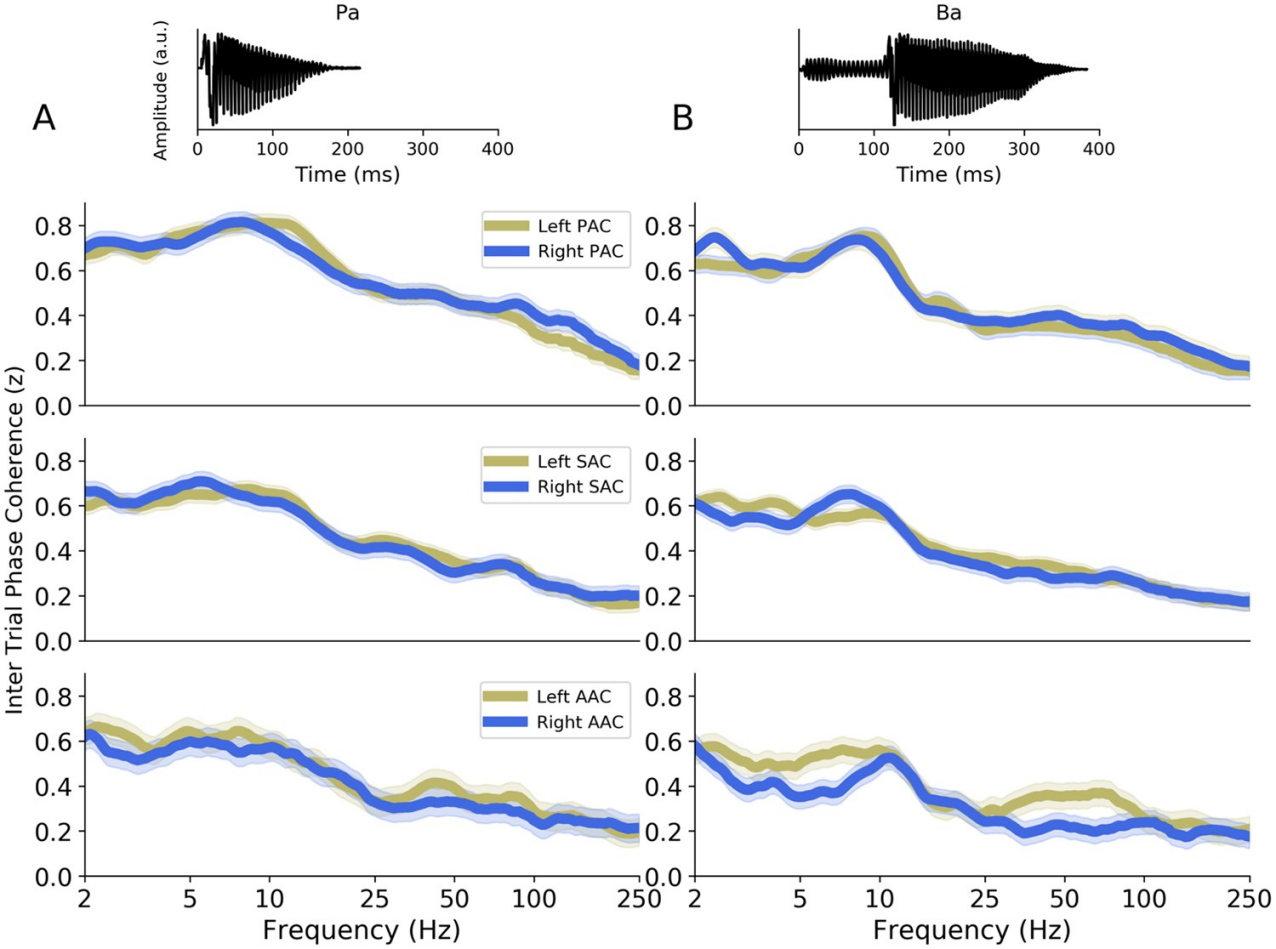
Evoked activity in response to French syllables /pa/ and /ba/ in the different auditory areas. (A–B) Top panels: acoustic waveform of the syllables. Lower panels: interhemispheric comparison of the ITPC spectra in response to the syllables (A) /pa/ and (B) /ba/, in PAC, SAC, and AAC. AAC, association auditory cortex; a.u., arbitrary unit; ITPC, intertrial phase coherence; PAC, primary auditory cortex; SAC, secondary auditory cortex.

To evaluate the interaction between stimulus and neural dynamics, the same analysis was carried on data recorded on the same patients during presentation of syllables (French /ba/ and /pa/; Fig 6). Importantly, these stimuli are characterized by more complex spectrotemporal dynamics than transient pure tones and carry linguistic information. Accordingly, we observed that the ITPC spectral response profile differed between pure tones and /ba/ and /pa/ stimuli, as predicted. Responses to syllables yielded less prominent and specific spectral peaks in the different ROIs, even in the latter stages of auditory processing (AAC). The maximum neural activity in response to syllable presentation was in the low frequency range (<20 Hz) and did not change across ROIs.

We extracted for each patient, ROI, and syllable type (/ba/ or /pa/) the 2 highest local maxima of the ITPC spectrum. Overall, the peak frequencies were similar across ROIs and syllable types, with ITPC spectra dominated by a main peak in the delta/theta range (around 4 Hz), with a secondary peak in the beta range (around 18 Hz). Crucially, the frequency of the 2 main ITPC peaks did not differ significantly across hemispheres in PAC, SAC, or AAC (unpaired Mann–Whitney U tests: all *p*-values > 0.09).

**Fig 7:**
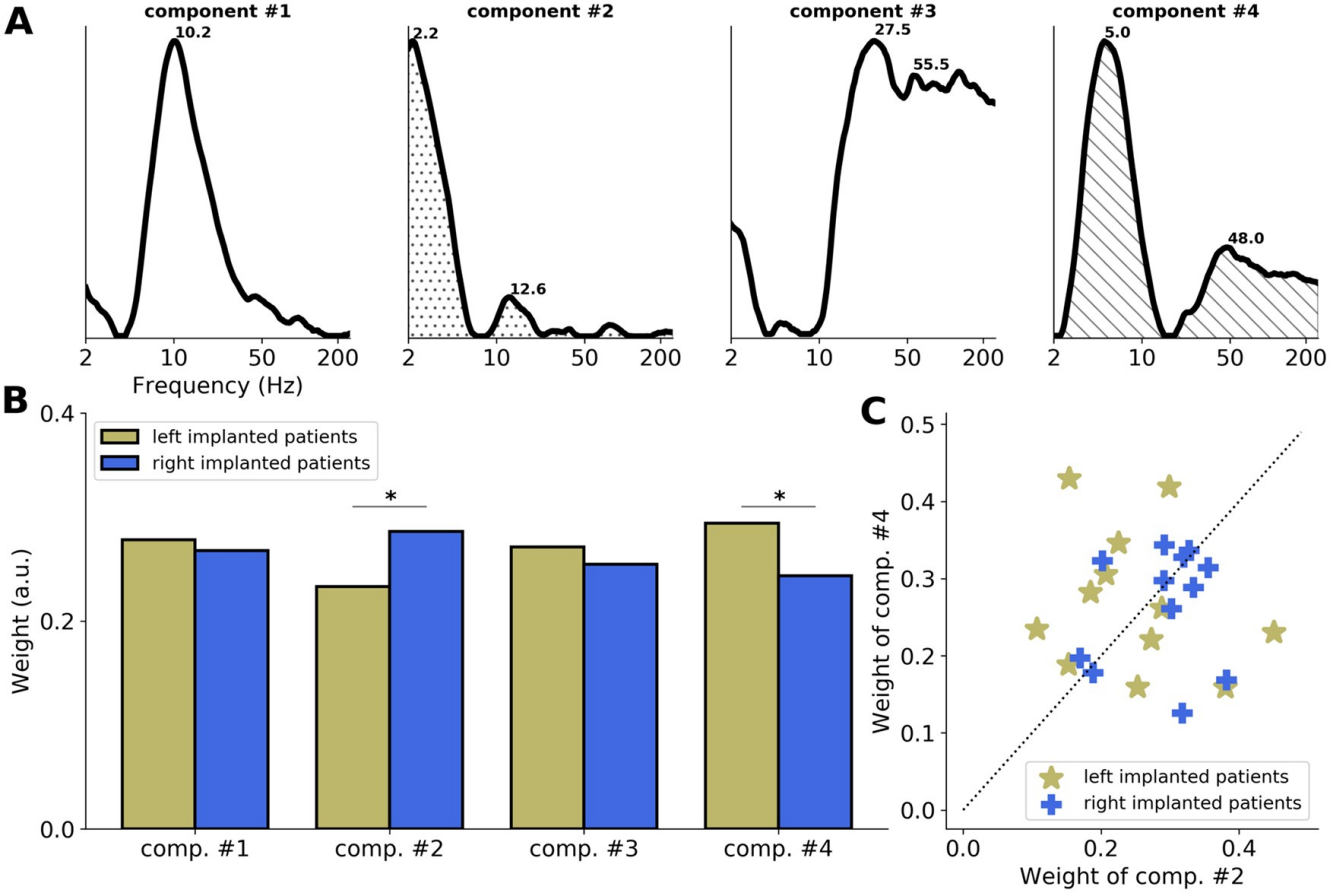
Main spectral components of the AAC response to syllables. The NMF method applied to the ITPC spectra in response to syllables (French /ba/ and /pa/ merged) of the entire set of electrode contacts implanted in AAC (left and right combined; *n* = 24). (A) Four main ITPC spectral components estimated with the NMF. Numbers indicate the main frequency peaks of each component. Components #2 and #4 are, respectively, characterized by a “delta/beta” (around 2/13 Hz) and “theta/gamma” (around 5/48 Hz) pattern, while components #1 and #3 have a spectral profile specific to syllable presentation. (B) Respective contribution (relative weight) of components #2 and #4 to left and right AAC data. Stars indicate significant differences (unpaired *t* tests, *p* < 0.05). (C) Respective contribution of components #2 (x-axis) and #4 (y-axis) to individual data. Patients are sorted according to the hemisphere of implantation (yellow: left; blue: right). The dashed diagonal indicates equal weights. AAC, association auditory cortex; a.u., arbitrary unit; ITPC, intertrial phase coherence; NMF, non-negative matrix factorization.

To better understand this result, we analyzed the entire set of electrode contacts implanted in AAC and performed an NMF clustering approach (as previously described). We extracted the 4 main ITPC spectral components, which altogether explained 55% of the variance of the data set. These components were characterized by 4 different spectral profiles (Fig 7A). While components #2 and #4 were, respectively, characterized by a “delta/beta” (around 2/13 Hz) and “theta/gamma” (around 5/48 Hz) pattern, components #1 and #3 had, respectively, an alpha (around 10 Hz) and gamma (>25 Hz) spectral profile, which were not observed in the responses to pure tones. These latter components can thus be interpreted as specific to syllables (i.e., more acoustically complex and/or linguistic) stimuli processing.

We evaluated the relative weight of these components on left or right AAC responses (Fig 7B). Across electrode contacts, we observed that 2 out of the 4 components were asymmetrically distributed. While components #1 (alpha) and #3 (gamma) were not significantly lateralized (unpaired *t* tests: component #1: *t* = 0.7, *p* = 0.49; component #3: *t* = 1.5, *p* = 0.14), component #4 (theta/gamma pattern) was significantly more present in left than right AAC (*t* = 3.8, *p* < 0.001), and component #2 (delta/beta pattern) was significantly more present in right than left AAC (*t* = 4.0, *p* < 0.001). We finally estimated whether this interaction was robust at the individual level by extracting the contribution (relative weight) of components #2 and #4 to the individual data (Fig 7C). We observed that only 54% of the patients implanted in AAC had a response profile predominantly composed of the expected spectral component (7/12 dominated by a theta/gamma component [#4] in left AAC; 6/12 dominated by a delta/beta component [#2] in right AAC).

This set of analyses first reveals that during perception of more complex stimuli—which carry a spectrotemporal dynamics and/or linguistic information—the evoked neural response is more heterogeneous, in particular with the emergence of alpha and gamma activity bilaterally in AAC (Fig 7). Second, it shows that the functionally asymmetric response observed during pure tone processing is also present during syllable processing, as revealed by the clustering analysis, with predominant theta/gamma (around 5/48 Hz) activity in left AAC and predominant delta/beta (around 2/13 Hz) activity in right AAC. However, this asymmetry is less salient, as evidenced by the absence of visible differences in the raw ITPC spectral profiles (Fig 6) and the difficulty to observe it at the individual level (Fig 7C).

## Discussion

Derived from intracranial recordings from 96 epileptic patients and using transient auditory stimulation with tones and syllables, this study aimed to characterize the intrinsic timescales of auditory information processing at 3 stages of the auditory cortical hierarchy. The spatial and temporal precision offered by stereotactic electroencephalography (SEEG) recording enables a meticulous description of the neural dynamics at each anatomic stage. Using transient acoustic stimulation allowed us to probe stereotyped evoked neural responses that uncover the intrinsic dynamics of the recorded areas. Using syllables, which are characterized by more complex spectrotemporal dynamics, allowed us to further investigate the interaction between stimulus and neural dynamics.

Our results reveal first, that the early cortical stages of auditory processing (PAC, SAC), when acoustically stimulated with transient pure tone stimuli, show characteristic bimodal spectral profiles. These functional responses were characterized by a main spectral peak in the theta range (around 4–8 Hz), with a secondary peak in the beta/gamma range (around 15–40 Hz; Fig 3). This finding, obtained with high-resolution data on a large cohort of patients, is consistent with previous findings obtained with intracranial recordings on a single case [18]. Moreover, the presence of 2 concomitant time constants in the dynamics of the evoked response is a strong evidence in favor of the AST framework, in which the auditory system makes use of a two-timescale processing mode to perceptually sample acoustic dynamics [9,13]. This specific bimodal neural signature also corroborates physiological descriptions of a natural interaction between low and high oscillatory frequencies (phase-amplitude coupling) at rest and during auditory stimulation at the level of local neural ensembles [45]. No hemispheric difference is apparent in core auditory regions, as evidenced by the similar bimodal spectral pattern elicited in left and right PAC and SAC. This is in contradistinction with previous findings describing functional asymmetries in core auditory areas [16–18,21,22,30–32]. One limitation of SEEG recording is the absence of whole-brain coverage. In particular, our functional characterization of early cortical auditory processing was limited to the posteromedial portion of Heschl’s gyrus (PAC) and the lateral posterior STG (SAC; Fig 1). Hence, we cannot exclude the presence of a functional asymmetry in core auditory regions, notably in the lateral portion of Heschl’s gyrus and the planum polare [46], which we did not sample. However, our results are compatible with current models of the functional organization of the core auditory cortex, which report relatively weak functional hemispheric differences [47].

Second, our results show the emergence of a strong functional asymmetry at the level of AAC. Using stimulation with short acoustic transients, we observed predominant theta/gamma (around 5/45 Hz) activity in left AAC and predominant delta/beta (around 2.5/16 Hz) activity in right AAC (Figs 2–5). Importantly, the clustering approach revealed that in AAC, the evoked responses to a brief acoustic stimulation are principally composed of these theta/gamma and delta/beta bimodal spectral patterns (Fig 5). Given the limitations/constraints imposed by the nature of the population under study, the emergence of a functional asymmetry in AAC was mostly demonstrated across patients at the group level. We, however, show that for 80% (16/20) of the patients implanted in AAC that had a typical language lateralization in the left hemisphere, the response profile was predominantly composed of the expected spectral components (theta/gamma in left AAC and delta/beta in right AAC). Moreover, our data set included a patient implanted bilaterally in AAC for whom we observed similar response profiles (Fig 4C), overall highlighting the robustness of the results at the individual level. Of note, because only 4 out of 24 patients implanted in AAC had a nontypical language lateralization that was also complex, reflecting a bilateral organization of language functions, it is difficult to estimate whether the observed functional asymmetry supports the lateralization of language functions.

Third, our results indicate that whereas a striking functional asymmetry in the higher auditory regions (AAC) is visible during brief acoustic stimulation, it is somehow obscured but nonetheless still present during the processing of more acoustically complex stimuli such as syllables. Indeed, the methodology used in the present study necessitates probing the brain with a short acoustic transient or impulse—i.e., a signal without any temporal dynamics. However, the functional asymmetry observed during pure tone processing in AAC is also present during syllable processing, as revealed by the clustering analysis (Fig 7B). This asymmetry is less salient, as evidenced by the absence of visible differences in the raw ITPC spectral profiles (Fig 6) and the difficulty to observe it at the individual level (Fig 7C). One reason is that the evoked response to syllables is not only composed of theta/gamma and delta/beta bimodal spectral patterns but is also more heterogeneous, with the emergence of alpha and gamma activity (Fig 7A). Those complementary spectral modes may emerge from the elaborate interaction between the spectrotemporal acoustic features of the stimulus and the intrinsic neural activity and/or reflect the processing of linguistic (phonemic) information.

Most of our results—obtained with short acoustic transients and, to a lesser extent, with syllables—are in accordance with the main tenets of the original AST hypothesis, notably with point 1 (the observation of a bimodal spectral profile in all the areas investigated) and point 3 (the emergence of a functional asymmetry in association areas). However, they also reveal that this functional asymmetry does not simply correspond to a differential ratio of neural ensembles oscillating at theta and gamma rates (point 2) [6] but, in fact, corresponds to the involvement of distinct dynamics (theta/gamma versus delta/beta) in left and right hemispheres. Reframing the AST hypothesis after our findings could result in the following set of hypotheses:

1. The spectral profile of neural response in left AAC (but also bilateral PAC and SAC) could be linked to a recent model of coupled oscillators describing the sensory analysis of speech, in which low and high frequency oscillations operate in the theta and gamma ranges, respectively, and process in parallel acoustic information [48]. In this model, the tracking of slow speech fluctuations by theta oscillations and its coupling to gamma activity both appear as critical features for accurate speech encoding, underscoring the importance of a two-timescale processing mode for efficiently analyzing speech. Moreover, this model suggests that during speech perception, syllabic- and phonemic-scale computations operate in combination at a local cortical level of processing, which could correspond to the left AAC.
2. On the other hand, the presence of neural activity in the delta and beta ranges in right AAC is more puzzling. Previous studies claimed that parsing at the syllabic scale occurs bilaterally [49] or is even rightward lateralized [14,23]. However, the right auditory cortex is more sensitive to spectral than temporal modulations [27,28,50], and perception of prosody is a right-lateralized process [51]. Thus, our observation of a specific response dynamics in right AAC could be linked to neural mechanisms dedicated to the parsing of spectral acoustic dynamics. Prosodic phenomena at the level of intonation contours are an example of such a phenomenon; the successful perceptual analysis of spoken language requires the processing of the rhythmic and melodic variations in speech to gain knowledge about speaker’s emotions and intentions [51]. The delta intrinsic timescale observed in right AAC would be particularly well-suited to the segmentation of prosodic cues because they naturally unfold at 0.5–3 Hz, as also argued in a recent computational model [52].

Overall, our results shed light on the neurofunctional architecture of cortical auditory processing and in particular on the specific processing timescales of different cortical areas. These general mechanisms are thought to apply to general auditory as well as speech perception. By integrating our findings to the AST hypothesis, we would speculate that syllabic and phonemic information is segmented in parallel locally, through coupled theta and gamma oscillations, while right-lateralized processes such as intonation contour or prosody perception would be segmented by delta (and beta) oscillations. The methodology we employed here is only suited to transient stimuli because longer stimuli with a spectrotemporal dynamics impose strong temporal constraints on the neural activity, resulting in more heterogeneous and elaborate response profiles. It is thus an “intermediate” method between resting state and speech paradigms, allowing a more precise description of the natural dynamics at play throughout the auditory pathway.

## Materials and methods

### Ethics statement

The study was approved in accordance with the Declaration of Helsinki by the Institutional Review board of the French Institute of Health (IRB00003888). Patients provided written informed consent prior to the experimental session. Participation was voluntary, and none of these patients participated in a clinical trial.

### Participants

96 patients (46 females) with pharmacoresistant epilepsy took part in the study. They were implanted with depth electrodes for clinical purpose at the Hôpital de la Timone (Marseille). Their native language was French. Neuropsychological assessments carried out before SEEG recordings indicated that all patients had intact language functions and met the criteria for normal hearing. None of them had their epileptogenic zone including the auditory areas as identified by experienced epileptologists.

### Evaluation of language laterality

For each patient, hemispheric specialization of language functions was determined by a trained clinician on the basis of several clinical assessments. Those included 1) the correlation of language deficits during seizure and postictal periods, 2) a functional mapping of the regions associated to language impairment using direct electrical stimulations, and 3) a functional mapping of the regions producing gamma (>40 Hz) activity during a picture naming task [53]. Additionally, functional MRI, handedness, neuropsychological data, and, if necessary, a Wada test were also available to determine the hemispheric lateralization of language functions.

On the basis of this clinical information, we were able to classify patients into 3 groups: typical left-lateralized, atypical right-lateralized, and atypical complex (i.e., bilateral organization of language functions). For this latter group, it was difficult to determine for the different regions implicated in language processing (auditory, temporal, frontal) whether they had a typical or atypical organization.

### SEEG method

A full description of the SEEG method is provided in S1 Text. Briefly, SEEG is a type of presurgical investigation based on implantation of multiple intracerebral electrodes, suitable for all types of drug-resistant epilepsies. There is no "standard" electrode implantation, the position of electrodes being chosen according to individual clinical characteristics. The implantation is performed according to the Bancaud and Talairach stereotactic method [54], with most of the electrodes implanted orthogonally through the double talairach grid.

Patients were implanted with an average of 11 (range [3–20]) depth electrodes (0.8 mm), composed of 5–15 contacts. Contacts were 2 mm long and spaced by 1.5 mm. The number of contacts per patient was on average of 138 (range [45–256]). Out of 96 patients, 45 were implanted in only one of the investigated regions. Only 3/96 patients were implanted bilaterally, including 1 in the associative cortex.

Neural recordings were performed between 4 to 9 days after the implantation procedure. No sedation or analgesics drugs were used, and antiepileptic drugs were partially or completely withdrawn. Recordings were always acquired after more than 4 hours to the last seizure.

### Stimuli and paradigm

Two types of auditory stimuli were presented to patients in 2 separate sessions: 1) 30-ms–long pure tones, presented binaurally at 500 Hz or 1 kHz (with a linear rise and fall time of 0.3 ms) 110 times each, with an ISI of 1,030 (±200) ms; and 2) /ba/ or /pa/ syllables, pronounced by a French female speaker (Fig 4, top) and presented binaurally 250 times each, with an ISI of 1,030 (±200) ms. These stimuli were designed for a clinical purpose in order to functionally map the auditory cortex [30,38,39]. During the 2 recording sessions, patients laid comfortably in a chair in a sound attenuated room and listen passively to the stimuli. Auditory stimuli were delivered from loudspeakers in front of the patients at a comfortable volume. Stimuli were presented in a pseudorandom order at a 44-kHz rate using E-prime 1.1 (Psychology Software Tools Inc., Pittsburgh, PA, USA).

### Functional localization of the auditory areas

Depth electrodes containing 5–15 contacts (see S1 Text) were used to perform the functional stereotactic exploration. The locations of the electrode implantations were determined solely on clinical grounds. To determine which auditory areas had been implanted, we relied on a functional localizer [30,38,39,42]. For each patient, AEPs in response to pure tones (500 Hz and 1 kHz merged) were used to functionally delineate the different auditory areas and to select the most relevant electrode contacts. AEPs were averaged over trials after epoching (−200 to 635 ms). A baseline correction was applied on each trial by computing a z-score relative to the −150 ms to 50 ms prestimulus time period. Epochs with artifacts and epileptic spikes were discarded by visual inspection prior to being averaged over trials. All contacts that elicited no significant response (<40 μV) were discarded.

The ROIs were functionally defined based on the presence of specific electrophysiological markers in the AEPs (early P20/N30, N/P50, and N/P 60–100) for PAC, SAC, and AAC (Fig 1) [30,38,39,42]. Among the 96 patients, respectively, 39, 40, and 12 had (at least) a contact in left PAC, SAC, and AAC, and 27, 29, and 12 had a contact in right PAC, SAC, and AAC. For each patient and ROI, the most responsive contact (i.e., the contact with the largest AEP) was selected for subsequent analyses when multiple contacts were present in the functional ROI. In a complementary set of analysis centered on AAC, we exploited all the contacts present in the ROI to perform an unsupervised clustering analysis.

### SEEG recordings

SEEG signals were recorded at a sampling rate of 1,000 Hz using a 256-channel BrainAmp amplifier system (Brain Products GmbH, Munich, Germany) and bandpass filtered between 0.3 and 500 Hz. A scalp electrode placed in Fz was used as the recording reference. SEEG data were epoched between −5 s to 5 s relative to stimulus onset (either pure tones or syllables). Such a long temporal window for epoching allowed a more precise frequency resolution for time-frequency analysis. Epochs with artifacts and epileptic spikes were discarded by visual inspection. Data were referenced into a bipolar montage by subtracting activity recorded at each contact of interest from activity acquired at its closest neighbor site within the same electrode.

### ITPC analysis

Trial-by-trial time-frequency analysis was carried out in a frequency range of 2–250 Hz (logarithmically spaced). The time-resolved spectral decomposition was performed by applying a Morlet wavelet transform to the data using the MNE-python function *time_frequency*.*tfr_morlet* (n_cycles = 7 and frequency steps = 100) [55]. This function also returns the ITPC, which is an estimate across trials of the time-frequency profile of the evoked activity. This measure quantifies the stereotypicality (i.e., consistency across trials) of the response per frequency, which highlights the intrinsic co-occurring time constants comprising the dynamical evoked response. For each time and frequency point, an ITPC value close to 0 reflects low phase consistency across trials, whereas an ITPC value of 1 reflects a perfect phase consistency across trials. The ITPC spectrum was then computed by averaging over time the ITPC values within a time window of interest designed to encompass one oscillatory cycle. Hence, the time window varies across frequencies (for example, 0–500 ms at 2 Hz or 0–20 ms at 50 Hz; see black overlays in Fig 2A).

To investigate the frequencies at which the evoked activity was maximal and to be able to compare them across ROIs and patients, the resulting ITPC spectra were normalized across frequencies (z-score per patient and ROI). Finally, the 2 main peaks of each ITPC spectrum (each ROI and patient) were extracted. Automatic peak detection of the 2 highest noncontiguous local maxima was performed by use of the function find_peaks from the python package scipy.signal (minimal distance between peaks = 22 points; prominence of peaks = 0.01).

### LOOCV

An LOOCV was performed on all ITCP spectra from (left and right) AAC to assess whether the implantation hemisphere (left or right) could be predicted from the spectral response profile of the region to a brief auditory stimulation. In brief, for each patient implanted in AAC, we estimated the MSE (i.e., the error of fit) between the individual ITPC spectrum and both (1) the group-level ITPC pattern from all other patients implanted in the same hemisphere and (2) the group-level ITPC pattern from all patients implanted in the opposite hemisphere. For each of these 2 measures, we determined an index of similarity, computed as the squared inverse of the MSE. Finally, we compared these 2 indices of similarity to evaluate whether a patient’s ITPC spectrum was more similar to the group-level ITPC pattern of the same hemisphere or to the group-level ITPC pattern of the opposite hemisphere. Identical values indicate equal similarity to both group-level ITPC patterns.

### NMF

All possible bipolar montages (i.e., 245) from the electrodes (i.e., 24) implanted in AAC were preprocessed using the same analysis pipeline as previously described. In brief, each bipolar montage’s time series was band-passed, epoched, transformed into ITPC, z-scored, and then time-averaged, resulting in individual ITPC spectra.

An NMF was conducted simultaneously on all resulting ITPC spectra. This clustering method was used to uncover prototypical ITPC patterns in an unsupervised manner. The non-negative input matrix V corresponding to the ITPC spectra [m frequencies × *n* contacts] is approximated as the matrix product of 2 non-negative matrix factors W [m × k] and H [k × n] (with k corresponding to the number of components of the decomposition) by optimizing the distance between V and W × H by using the squared Frobenius norm. The resulting matrix W contains a set of basis vectors that are linearly combined using the coefficients in H to represent the input data V. W gives meaningful “cluster centroids,” which are prototypical ITPC patterns [56]. H represents the weight matrix, or clusters membership. It is the estimate of the relative contribution of each component to individual ITPC spectra. The function decomposition.NMF() from the scikit learn python package was used to compute the NMF with a number of 4 components (k = 4) [57].

The proportion of variance explained (r2) by the 4 components of the NMF was computed in a cross-validation scheme. The data set was split into 2 subsets, training and testing, allowing fitting the NMF model on the training data (80% of the entire data set) in order to make predictions on the test data (20%).

### Statistical procedures

All analyses were performed at the level of individual electrodes contacts (bipolar montages) before applying standard nonparametric statistical tests at the group level (unpaired nonparametric Wilcoxon–Mann–Whitney tests or parametric *t* tests).

### Code availability

Codes to reproduce the results and figures of this manuscript are available on GitHub: https://github.com/DCP-INS/asymmetric-sampling.

## Supporting information

S1 Fig Individual examples of ITPC spectra in response to pure tones (0.5 kHz and 1 kHz merged) from 18 patients implanted in different auditory areas (yellow: Left hemisphere; blue: Right hemisphere). Dashed vertical lines indicate the 2 highest noncontiguous local maxima (black: first peak; gray: second peak). ITPC, intertrial phase coherence.
(TIF)

S2 Fig ITPC spectra in response to (A) 0.5 kHz and (B) 1 kHz pure tones in the different auditory areas. Interhemispheric comparison of the ITPC spectra in PAC, SAC, and AAC. Shaded areas indicate SEM. AAC, association auditory cortex; ITPC, intertrial phase coherence; PAC, primary auditory cortex; SAC, secondary auditory cortex.
(TIF)

S1 Text Clinical and methodological information on SEEG recordings. SEEG, stereotactic electroencephalography.
(DOCX)

## Abbreviations

AAC: association auditory cortex
AEP: auditory evoked potential
AST: asymmetric sampling in time
ERP: event-related potential
ITPC: intertrial phase coherence
LOOCV: leave-one-out cross validation
MNI: Montreal Neurological Institute
MSE: mean squared error
NMF: non-negative matrix factorization
PAC: primary auditory cortex
ROI: region of interest
SAC: secondary auditory cortex
SEEG: stereotactic electroencephalography
STG: superior temporal gyrus
