# Supplementary figures and images for: Asymmetric sampling in human auditory cortex reveals spectral processing hierarchy
Source: PLoS Biol. 2020 Mar 2;18(3):e3000207. doi: 10.1371/journal.pbio.3000207 (PMC7067489; doi:10.1371/journal.pbio.3000207)

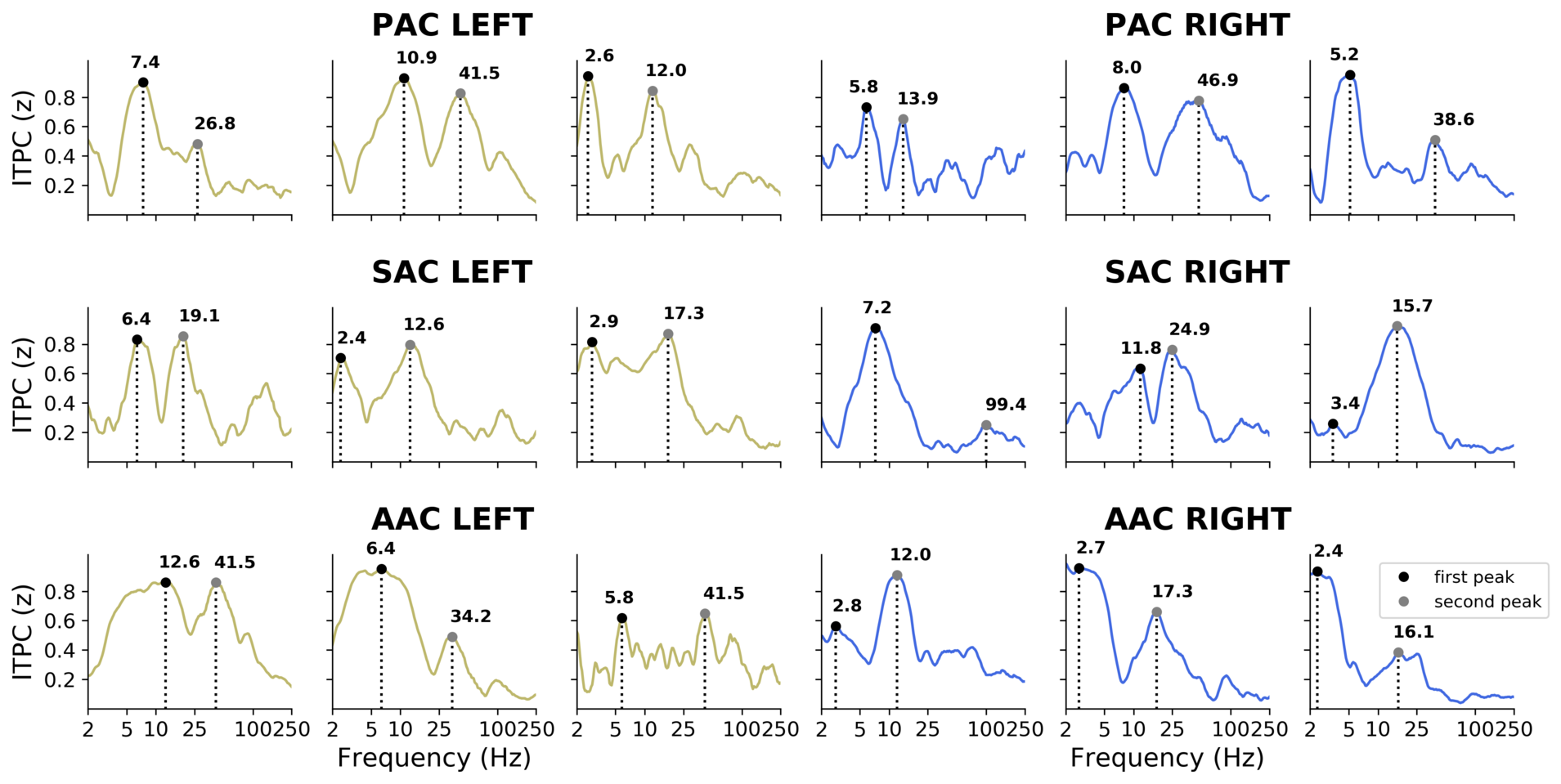

Supplement: S1 Fig — Dashed vertical lines indicate the 2 highest noncontiguous local maxima (black: first peak; gray: second peak). ITPC, intertrial phase coherence. (TIF) [file pbio.3000207.s001.tif]

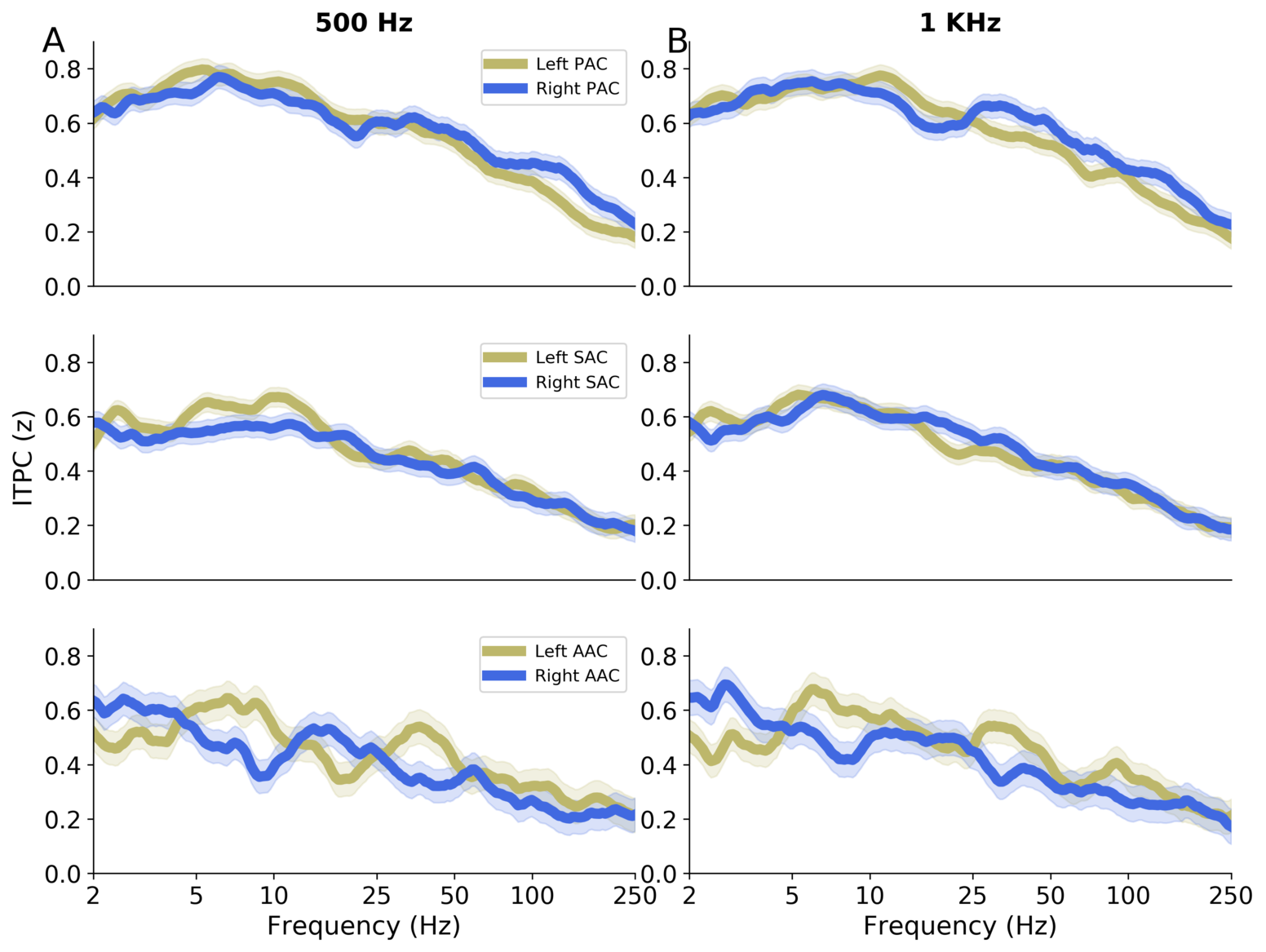

Supplement: S2 Fig — Interhemispheric comparison of the ITPC spectra in PAC, SAC, and AAC. Shaded areas indicate SEM. AAC, association auditory cortex; ITPC, intertrial phase coherence; PAC, primary auditory cortex; SAC, secondary auditory cortex. (TIF) [file pbio.3000207.s002.tif]
