# Supplementary material for: Asymmetric sampling in human auditory cortex reveals spectral processing hierarchy
Source: PLoS Biol. 2020 Mar 2;18(3):e3000207. doi: 10.1371/journal.pbio.3000207 (PMC7067489; doi:10.1371/journal.pbio.3000207)
Supplement: S1 Text — SEEG, stereotactic electroencephalography. (DOCX) [file pbio.3000207.s003.docx]

**Supporting information**

**The stereotactic EEG (SEEG) method.** SEEG has been for fifty years (developed by Jean Talairach) a type of presurgical investigation suitable for all types of drug-resistant epilepsies. SEEG is based on implantation of multiple intracerebral electrodes in order to record and map the epileptogenic and propagation networks. The SEEG method includes recording of spontaneous seizures, recording of neural activity during sensory stimulation, and direct cortical electrical stimulation to trigger seizures (part of the localization process) and perform functional mapping in the same areas (to evaluate possible contraindications due to sensory or cognitive impairment such as language deficits).

**Strategy of implantation.** There is no "standard" electrode implantation. Implantation strategy depends only on the hypotheses made about the anatomical location of the epileptogenic zone (EZ), with the aim of defining subsequent cortectomy. The hypotheses of the likely EZ localization are based on (phase I) non-invasive pre-surgical assessment including detailed clinical history, surface video-electroencephalographic (EEG) recording, MRI, and 18FDG-PET scanner. Electrode positions are therefore not standardized across patients, but chosen according to individual clinical characteristics.

One of the most frequent regions to be explored is the perisylvian region in order to know for instance if the patient's epilepsy is temporal, temporo-perisylvian, or perisylvian (the main cause of surgical failure in temporal epilepsy is a misdiagnosis of perisylvian epilepsies). Perisylvian epilepsy can be localized in insular cortex, frontal opercular, parietal opercular, temporal opercular cortices and superior temporal and supramarginal gyri. In this region, electrodes are implanted orthogonally to the cortical surface in order to record along one single electrode both posterior insula, tip of Heschl's gyrus and planum temporale, for example. Another trajectory is to implant more anteriorly the superior temporal gyrus and the ventral insula. The perisylvian region is also implanted in the hypothesis of inferior parietal lobule, pericentral, or ventral prefrontal/premotor epilepsy. The transverse gyrus (Heschl's gyrus, which includes the auditory cortex) is critically important because of its connections with lower central regions and the inferior frontal gyrus: it is a pathway through which seizures from the temporal pole and the anterior superior temporal gyrus generalize.

**Implantation procedure.** The implantation of electrodes is performed according to the Bancaud and Talairach stereotactic method [[1]](http://f1000.com/work/citation?ids=7631015&pre=&suf=&sa=0). The stereotaxic localization and the implantation of the intracerebral electrodes are done at the same time. The Leksell frame is fixed percutaneously, and MRI and cerebral angiography are obtained. Then, a catheter cerebral angiogram is obtained in telemetric and stereotaxic conditions, and trajectories are planned by looking at both 3D T1-weighted (T1W) MR and brain vasculature. All the anatomic information is merged in the stereotaxic space. Most of the electrodes are orthogonally implanted through the double talairach grid. The entry point of the electrodes is determined on the basis of the disposition of the vessels in the area. Finally, electrodes are implanted percutaneously by twist drill, and fixed using anchor bolts.

**Post-implantation procedure.** A post-operative electrode implantation computerized tomography (CT) scan is done to verify the absence of bleeding and the location of each recording lead. Following this, intracerebral electrodes are removed and an MRI scan is performed, permitting to visualise each electrode’s trajectory. Finally, CT-scan/MRI data fusion is performed to locate each contact along the electrode trajectory. The fusion can be done with several software: BrainVISA/Anatomist, medInria, Gardel [[2–4]](http://f1000.com/work/citation?ids=7630824,7640386,5586085&pre=&pre=&pre=&suf=&suf=&suf=&sa=0,0,0).

**Potential impact of drugs on neural recordings.** Neural recordings are performed between 4 to 9 days after the implantation procedure. No sedation or analgesics drugs are used. In general, a partial or complete withdrawal of antiepileptic drug is done prior to the beginning of the exploration. However, the level of medication is adjusted on an individual basis, as a function of the seizure type. Recordings are always acquired after more than 4 hours to the last seizure.

**Localisation of electrode contacts in the current dataset.** The 96 patients of our study were implanted with an average of 11 (median: 11; range [3-20]) depth electrodes (0.8 mm; Alcis, Besançon, France). Electrodes had between 5 and 15 contacts. Contacts were 2 mm long and spaced from each other by 1.5 mm. The number of contacts per patient was on average of 138 (median: 124; range [45-256]). The number of contacts per region depended on the implantation location and the nature of the region. More specifically, more associative regions are larger than primary ones. Thus, the primary auditory cortex was implanted with up to 3 contacts, the secondary cortex with up to 5 contacts, and the associative cortex with up to 15 contacts. Out of the 96 implanted patients, 45 were implanted in only one of the investigated regions. Most of the patients implanted in more than one region were implanted in the primary and secondary auditory cortex of the same hemisphere. Only 3 patients were implanted bilaterally, and only 1 patient was implanted bilaterally in the associative cortex.

**REFERENCES**

[1. Talairach J, Bancaud J, Bonis A, Szikla G, Tournoux P. Functional stereotaxic exploration of epilepsy. Stereotact Funct Neurosurg. 1962;22: 328–331. doi:10.1159/000104378](http://f1000.com/work/bibliography/7631015)

[2. Rivière D, Geffroy D, Denghien I, Souedet N, Cointepas Y. BrainVISA: an extensible software environment for sharing multimodal neuroimaging data and processing tools. Neuroimage. 2009;47: S163. doi:10.1016/S1053-8119(09)71720-3](http://f1000.com/work/bibliography/7630824)

[3. medInria. In: med.inria [Internet]. [cited 6 Feb 2019]. Available: http://med.inria.fr/](http://f1000.com/work/bibliography/7640386)

[4. Medina Villalon S, Paz R, Roehri N, Lagarde S, Pizzo F, Colombet B, et al. EpiTools, A software suite for presurgical brain mapping in epilepsy: Intracerebral EEG. J Neurosci Methods. 2018;303: 7–15. doi:10.1016/j.jneumeth.2018.03.018](http://f1000.com/work/bibliography/5586085)
